# *Shutdown corner*, a large deletion mutant isolated from a haploid mutagenesis screen in zebrafish

**DOI:** 10.1093/g3journal/jkab442

**Published:** 2021-12-23

**Authors:** Macaulie A Casey, Jonathon T Hill, Kazuyuki Hoshijima, Chase D Bryan, Suzanna L Gribble, J Thomas Brown, Chi-Bin Chien, H Joseph Yost, Kristen M Kwan

**Affiliations:** 1 Department of Human Genetics, University of Utah, Salt Lake City, UT 84112, USA; 2 Department of Cell Biology and Physiology, Brigham Young University, Provo, UT 84602, USA; 3 Department of Molecular Genetics and Microbiology, University of Florida, Gainesville, FL 32611, USA; 4 Department of Biological Sciences, University of Pittsburgh, Pittsburgh, PA 15260, USA; 5 Department of Biomedical Informatics, Vanderbilt University, Nashville, TN 37203, USA; 6 Department of Neurobiology and Anatomy, University of Utah, Salt Lake City, UT 84112, USA

**Keywords:** zebrafish, deletion, haploid screen, optic cup morphogenesis, locomotion, muscle, vasculature

## Abstract

Morphogenesis, the formation of three-dimensional organ structures, requires precise coupling of genetic regulation and complex cell behaviors. The genetic networks governing many morphogenetic systems, including that of the embryonic eye, are poorly understood. In zebrafish, several forward genetic screens have sought to identify factors regulating eye development. These screens often look for eye defects at stages after the optic cup is formed and when retinal neurogenesis is under way. This approach can make it difficult to identify mutants specific for morphogenesis, as opposed to neurogenesis. To this end, we carried out a forward genetic, small-scale haploid mutagenesis screen in zebrafish (*Danio rerio*) to identify factors that govern optic cup morphogenesis. We screened ∼100 genomes and isolated *shutdown corner* (*sco*), a mutant that exhibits multiple tissue defects and harbors a ∼10-Mb deletion that encompasses 89 annotated genes. Using a combination of live imaging and antibody staining, we found cell proliferation, cell death, and tissue patterning defects in the *sco* optic cup. We also observed other phenotypes, including paralysis, neuromuscular defects, and ocular vasculature defects. To date, the largest deletion mutants reported in zebrafish are engineered using CRISPR-Cas9 and are less than 300 kb. Because of the number of genes within the deletion interval, *shutdown corner* [*Df(Chr05:sco)^z207^*] could be a useful resource to the zebrafish community, as it may be helpful for gene mapping, understanding genetic interactions, or studying many genes lost in the mutant.

## Introduction

The formation of three-dimensional organ structures, for which structure and function are inextricably tied, requires precise coordination of cell movements and genetic regulation. Disrupting these processes can impair morphogenesis, leading to perturbed morphology and potential functional defects. The embryonic eye is an excellent morphogenetic model, as disruptions to optic cup formation can lead to visual deficits independent of neurogenesis. Optic cup morphogenesis is well-conserved across vertebrates; studies from salamander to mouse have uncovered many cell behaviors that shape the optic cup (Fuhrmann 2010; Sinn and Wittbrodt 2013; Kwan 2014; Bazin-Lopez et al. 2015; Martinez-Morales et al. 2017; Cavodeassi 2018; Casey et al. 2021). However, the genetic network which directs these behaviors remains poorly understood.

Zebrafish as a model system offers many advantages for studying early eye morphogenesis. The optic cup forms rapidly between 12 and 24 hours post fertilization (hpf); rapid development and optical clarity enable live imaging to visualize cell movements *in vivo* (Picker et al. 2009; KWan et al. 2012; Heermann et al. 2015; Nicolás-Pérez et al. 2016; Sidhaye and Norden 2017). Zebrafish are also a powerful genetic system, with both haploid and diploid genetic screening strategies (Walker 1999; Westerfield 2000; Patton and Zon 2001). Forward genetic screens have uncovered a wealth of genes governing embryogenesis (Driever et al. 1996; Haffter et al. 1996). Several of these screens examined the eye and assessed gross morphology from 1 to 5 days postfertilization (dpf), but many phenotypes were classified as “small eyes,” which could arise from a variety of developmental defects.

Screens specific for eye development have examined a number of developmental processes. Visual behavior and axon pathfinding were assayed at 5 dpf, after retinal neurogenesis (Brockerhoff et al. 1995; Baier et al. 1996; Karlstrom et al. 1996; Neuhauss et al. 1999). Earlier stages were also examined, uncovering defects in eye field induction (Heisenberg et al. 1996) or general abnormalities in eye and lens development (Driever et al. 1996; Haffter et al. 1996; Malicki et al. 1996; Fadool et al. 1997; Gross et al. 2005). Still others identified pigment defects (Rawls et al. 2003), or specific defects in eye morphogenesis, like coloboma (Lee et al. 2012). However, no prior eye development screens have focused specifically on the process of optic cup morphogenesis, where primary phenotyping would occur at 24 hpf.

To identify genes specifically involved in optic cup morphogenesis, we undertook a small-scale, haploid mutagenesis screen in zebrafish. We isolated one mutant, *shutdown corner* (*sco*), which exhibits a novel eye phenotype among other defects. Combining bulk RNA-sequencing and computational analysis, we determined *sco* harbors a ∼10-Mb deletion on chromosome 5 encompassing 89 annotated genes. Here, we characterize various phenotypes in *sco* mutants and report the list of deleted genes. Deletions of this size are uncommon in zebrafish; to date, only engineered deletions under 300 kb have been reported (Hoshijima et al. 2019; Kim and Zhang 2020; Tromp et al. 2021). The extent of deleted genes in *shutdown corner* [*Df(Chr05:sco)^z207^*] may make this mutant a useful resource for the zebrafish community.

## Materials and methods

### Zebrafish husbandry and transgenic lines

All zebrafish (*Danio rerio*) husbandry was performed under standard conditions in accordance with the University of Utah Institutional Animal Care and Use Committee (IACUC) Protocol approval (Protocol # 21-01007). Embryos (AB strain) were raised 28.5–29.5°C and staged according to time post fertilization and morphology (Kimmel et al. 1995). When necessary, melanization was prevented with the treatment of 0.003% 1-phenyl-2-thiourea (Sigma-Aldrich, P7629). Transgenic alleles used were: *Tg(bactin2:EGFP-CAAX)^z200^* (Gordon et al. 2018); *Tg(kdrl:mCherry-ras)^s896^* (Chi et al. 2008); *Tg(-8.0cldnb:lyn-EGFP)^zf106^* (Haas and Gilmour 2006).

For genotyping, genomic DNA was extracted from single embryos or adult fins, incubated in 0.05 M NaOH at 95°C for 30 min, then neutralized in 1 M Tris. The *Df(Chr05:sco)^z207^* locus was identified by PCR, using primers either flanking or positioned within the deletion interval (Fig. 6d): SCO_F: 5′-CAGTGTGTGTTACTGCTTACACAACATG-3′; WT_R: 5′-GCACTTTTCTACTCACAACACTTGTTTTTGAAG-3′; MUT_R: 5′-GGGAAATATTTGGCAAAGAATCAAATTTTCAAAGCC-3′. The wild-type amplicon is 774 bp and the *sco* amplicon is 497 bp, while heterozygotes yield two bands. “Mutants” were confirmed homozygous *sco* mutants, while “wild-type siblings” were confirmed homozygous wild-type or heterozygous carriers.

### Haploid mutagenesis screen

For mutagenesis, F_0_ males were generated by fasting AB males and then placing them in 3 mM ENU (*N*-ethyl-*N*-nitrosourea; Sigma-Aldrich, N3385) for 1 h. This treatment was repeated weekly for 3–6 weeks; then, the fish were allowed to recover for 4 weeks before crossing them (protocol based on Mullins et al. 1994; Solnica-Krezel et al. 1994). For in vitro fertilization and production of haploids: wild-type TL males were anesthetized in tricaine (Sigma-Aldrich, E10521; 400 mg/100 ml, diluted further into 100 ml system water) and squeezed to cause sperm release. Sperm were collected in ice cold Hank’s solution (recipes in The Zebrafish Book, Westerfield 2000) and subjected to UV crosslinking using a Stratalinker. Female F_1_s were anesthetized in tricaine and squeezed for eggs in a 35-mm plastic Petri dish. Eggs were fertilized with the UV-treated sperm by adding the sperm suspension in Hank’s solution to the eggs, followed by system water (Streisinger et al. 1981; Westerfield 2000; Kroeger et al. 2014). Typically, ∼75% of the eggs were fertilized. Haploidization was assessed at 24 hpf based on presence of haploid phenotypes, including impaired axis extension and duplicated otic vesicle (Westerfield 2000). Phenotypic screening for defective optic cup morphogenesis was performed at 24 hpf, via examination on an Olympus SZX16 dissecting stereomicroscope.

### Bulk RNA-sequencing and MMAPPR analysis

Total RNA was harvested from pools of sibling and mutant embryos (30 embryos each), at 3 dpf. The embryonic phenotype was identified at 24 hpf using the eye phenotype, and further confirmed at 3 dpf evaluating embryos for loss of locomotion. TRIzol reagent (Invitrogen, 15596026) was used for RNA isolation, according to the manufacturer’s standard protocol (1 ml TRIzol for 30 embryos). Samples were sequenced through the University of Utah High-Throughput and Bioinformatic Analysis Shared Resource. The resulting sequences were aligned to the zebrafish genome (GRCz11) using hisat2 version 2.1 (Kim et al. 2015). Single-nucleotide polymorphisms were identified and allele frequencies compared between phenotypic and wild-type pools using MMAPPR version 0.83 (Hill et al. 2013) with default settings. As MMAPPR identified linkage peaks surrounding a gap in the data, we suspected a large deletion. This was confirmed by comparing raw read counts between pools. Reads were aligned and assigned to genes using the Rsubread package (Liao et al. 2019). Resulting counts were then fitted by loess regression (span = 0.03) and plotted to compare read coverage between the wild-type pool and the phenotypic pool.

### Breakpoint mapping

The putative deletion identified by MMAPPR was first confirmed using PCR of genomic DNA for regions within and outside of the predicted deletion interval. Because RNAseq revealed transcripts that were affected by the deletion, PCR of genomic DNA, based on zebrafish genome assemblies, was used to “walk” closer to each end of the breakpoint until the breakpoint could be amplified and TOPO-TA cloned (Invitrogen, 450071). Once cloned, the precise breakpoint was identified using Sanger sequencing.

The list of deleted genes reported in Table 1 was exported from Ensembl using BioMart. The dataset included zebrafish genes from the GRCz11 genome build, located in the range of Chr5:42469846–51532989; only genes with a ZFIN ID were exported. For the user’s ease, we exported the gene stable ID, gene name, gene type, gene synonym, and gene description.

**Table 1.** Annotated genes in the *shutdown corner* deletion interval.

Gene stable ID	Gene name	Gene type	Strand	Gene description	Gene synonym
ENSDARG00000099531	pimr58	protein_coding	1	Pim proto-oncogene, serine/threonine kinase, related 58 [source: ZFIN; Acc: ZDB-GENE-060526-96]	si:ch211-207c6.9
ENSDARG00000004830	flot2a	protein_coding	−1	flotillin 2a [source: NCBI gene; Acc: 245698]	fb48a04
ENSDARG00000100662	cxcl11.1	protein_coding	−1	chemokine (C-X-C motif) ligand 11, duplicate 1 [source: NCBI gene; Acc: 798892]	CXCL-chr5d
ENSDARG00000075163	cxcl20	protein_coding	−1	chemokine (C-X-C motif) ligand 20 [source: ZFIN; Acc: ZDB-GENE-111004-2]	cxcl-c5c
ENSDARG00000029692	rufy3	protein_coding	1	RUN and FYVE domain containing 3 [source: ZFIN; Acc: ZDB-GENE-050327-58]	im:7148884
ENSDARG00000053021	grsf1	protein_coding	−1	G-rich RNA sequence binding factor 1 [source: ZFIN; Acc: ZDB-GENE-060825-196]	wu:fb62c04
ENSDARG00000009169	mob1ba	protein_coding	1	MOB kinase activator 1Ba [source: ZFIN; Acc: ZDB-GENE-040426-919]	mats2
ENSDARG00000003808	aqp3a	protein_coding	1	aquaporin 3a [source: ZFIN; Acc: ZDB-GENE-040426-2826]	wu:fa95h06
ENSDARG00000103167	si:dkey-245n4.2	protein_coding	−1	si:dkey-245n4.2 [source: ZFIN; Acc: ZDB-GENE-141216-258]	
ENSDARG00000010248	wdr54	protein_coding	1	WD repeat domain 54 [source: ZFIN; Acc: ZDB-GENE-040801-151]	zgc:100930
ENSDARG00000016868	rhobtb4	protein_coding	1	Rho related BTB domain containing 4 [source: ZFIN; Acc: ZDB-GENE-060315-11]	rhobtb2a
ENSDARG00000032482	si:dkey-40c11.2	protein_coding	1	si:dkey-40c11.2 [source: ZFIN; Acc: ZDB-GENE-060526-300]	cb540
ENSDARG00000031345	RTKN	protein_coding	−1	si:dkey-40c11.1 [source: ZFIN; Acc: ZDB-GENE-060531-142]	
ENSDARG00000026925	nos2a	protein_coding	1	nitric oxide synthase 2a, inducible [source: ZFIN; Acc: ZDB-GENE-040305-1]	inducible nitric oxide synthase a
ENSDARG00000018494	smn1	protein_coding	−1	survival of motor neuron 1, telomeric [source: NCBI gene; Acc: 30432]	fa12d01
ENSDARG00000067777	zgc:158640	protein_coding	1	zgc:158640 [source: ZFIN; Acc: ZDB-GENE-061215-15]	si:dkey-57m14.2
ENSDARG00000017571	mccc2	protein_coding	−1	methylcrotonoyl-CoA carboxylase 2 (beta) [source: ZFIN; Acc: ZDB-GENE-040426-2493]	si:dkey-57m14.1
ENSDARG00000102375	si:ch211-204c21.1	protein_coding	−1	si:ch211-204c21.1 [source: ZFIN; Acc: ZDB-GENE-030429-35]	sb:cb458
ENSDARG00000053091	DAB2	protein_coding	−1	si:ch211-204c21.1 [source: ZFIN; Acc: ZDB-GENE-030429-35]	sb:cb458
ENSDARG00000095369	zgc:112966	protein_coding	1	zgc:112966 [source: ZFIN; Acc: ZDB-GENE-050320-137]	
ENSDARG00000075126	TMEM8B	protein_coding	1	si:dkey-84j12.1 [source: ZFIN; Acc: ZDB-GENE-060526-342]	
ENSDARG00000094625	si:dkey-84j12.1	protein_coding	1	si:dkey-84j12.1 [source: ZFIN; Acc: ZDB-GENE-060526-342]	
ENSDARG00000094268	si:ch73-337l15.2	protein_coding	−1	si:ch73-337l15.2 [source: ZFIN; Acc: ZDB-GENE-041008-80]	im:7136138
ENSDARG00000013250	tars1	protein_coding	1	threonyl-tRNA synthetase 1 [source: ZFIN; Acc: ZDB-GENE-041010-218]	Tars
ENSDARG00000077298	gas1a	protein_coding	−1	growth arrest-specific 1a [source: ZFIN; Acc: ZDB-GENE-050302-155]	id:ibd5013
ENSDARG00000060093	dapk1	protein_coding	1	death-associated protein kinase 1 [source: ZFIN; Acc: ZDB-GENE-060526-177]	si:ch211-66i11.1
ENSDARG00000007836	ctsla	protein_coding	1	cathepsin La [source: ZFIN; Acc: ZDB-GENE-030131-106]	cb143
ENSDARG00000012366	fbp2	protein_coding	−1	fructose-1,6-bisphosphatase 2 [source: ZFIN; Acc: ZDB-GENE-040822-23]	zgc:101083
ENSDARG00000021366	fbp1a	protein_coding	−1	fructose-1,6-bisphosphatase 1a [source: ZFIN; Acc: ZDB-GENE-030131-7171]	fbp1l
ENSDARG00000060102	kank1a	protein_coding	1	KN motif and ankyrin repeat domains 1a [source: ZFIN; Acc: ZDB-GENE-060526-215]	ankrd15
ENSDARG00000007349	dmrt1	protein_coding	1	doublesex and mab-3 related transcription factor 1 [source: NCBI gene; Acc: 402923]	zgc:136676
ENSDARG00000035290	dmrt3a	protein_coding	1	doublesex and mab-3 related transcription factor 3a [source: NCBI gene; Acc: 450035]	Dmrt3
ENSDARG00000015072	dmrt2a	protein_coding	1	doublesex and mab-3 related transcription factor 2a [source: ZFIN; Acc: ZDB-GENE-990621-7]	dmrt2
ENSDARG00000008904	smarca2	protein_coding	1	SWI/SNF related, matrix associated, actin dependent regulator of chromatin, subfamily a, member 2 [source: ZFIN; Acc: ZDB-GENE-030131-5964]	wu:fa56c07
ENSDARG00000060127	adamts3	protein_coding	1	ADAM metallopeptidase with thrombospondin type 1 motif, 3 [source: ZFIN; Acc: ZDB-GENE-110223-1]	
ENSDARG00000089310	gc	protein_coding	1	GC vitamin D binding protein [source: ZFIN; Acc: ZDB-GENE-040718-307]	dbp
ENSDARG00000013730	slc4a4a	protein_coding	−1	solute carrier family 4 member 4a [source: ZFIN; Acc: ZDB-GENE-060526-274]	id:ibd2520
ENSDARG00000067795	ifngr2	protein_coding	−1	interferon gamma receptor 2 [source: ZFIN; Acc: ZDB-GENE-030131-5999]	crfb6
ENSDARG00000059963	polk	protein_coding	1	polymerase (DNA directed) kappa [source: ZFIN; Acc: ZDB-GENE-060526-137]	si:ch211-254o18.3
ENSDARG00000025866	ankdd1b	protein_coding	1	ankyrin repeat and death domain containing 1B [source: ZFIN; Acc: ZDB-GENE-060526-136]	si:ch211-254o18.2
ENSDARG00000059982	poc5	protein_coding	−1	POC5 centriolar protein homolog (Chlamydomonas) [source: ZFIN; Acc: ZDB-GENE-060526-135]	si:ch211-254o18.1
ENSDARG00000059997	sv2ca	protein_coding	1	synaptic vesicle glycoprotein 2Ca [source: ZFIN; Acc: ZDB-GENE-060526-233]	si:dkey-18p14.1
ENSDARG00000060010	iqgap2	protein_coding	1	IQ motif containing GTPase activating protein 2 [source: ZFIN; Acc: ZDB-GENE-030131-2878]	fc20f09
ENSDARG00000090524	f2rl2	protein_coding	−1	coagulation factor II (thrombin) receptor-like 2 [source: ZFIN; Acc: ZDB-GENE-110127-4]	par3
ENSDARG00000060012	f2r	protein_coding	1	coagulation factor II (thrombin) receptor [source: ZFIN; Acc: ZDB-GENE-060526-30]	PAR1-5A
ENSDARG00000057395	si:ch211-130m23.3	protein_coding	−1	si:ch211-130m23.3 [source: ZFIN; Acc: ZDB-GENE-060531-14]	
ENSDARG00000053159	zgc:110626	protein_coding	1	zgc:110626 [source: ZFIN; Acc: ZDB-GENE-050417-447]	im:7138190
ENSDARG00000095136	si:ch211-130m23.2	protein_coding	1	si:ch211-130m23.2 [source: ZFIN; Acc: ZDB-GENE-060531-13]	
ENSDARG00000098013	si:ch211-130m23.5	protein_coding	−1	si:ch211-130m23.5 [source: ZFIN; Acc: ZDB-GENE-131121-298]	
ENSDARG00000103515	vcana	protein_coding	1	versican a [source: ZFIN; Acc: ZDB-GENE-011023-1]	br146
ENSDARG00000089769	hapln1a	protein_coding	−1	hyaluronan and proteoglycan link protein 1a [source: ZFIN; Acc: ZDB-GENE-050302-175]	crtl1
ENSDARG00000093413	edil3a	protein_coding	−1	EGF-like repeats and discoidin I-like domains 3a [source: ZFIN; Acc: ZDB-GENE-060503-366]	edil3
ENSDARG00000093413	edil3a	protein_coding	−1	EGF-like repeats and discoidin I-like domains 3a [source: ZFIN; Acc: ZDB-GENE-060503-366]	si:dkey-84i7.1
ENSDARG00000104537	cox7c	protein_coding	−1	cytochrome c oxidase subunit 7C [source: ZFIN; Acc: ZDB-GENE-030131-8062]	wu:fj49c05
ENSDARG00000035535	rasa1a	protein_coding	1	RAS p21 protein activator (GTPase activating protein) 1a [source: ZFIN; Acc: ZDB-GENE-030131-4694]	fd52c05
ENSDARG00000007657	ccnh	protein_coding	−1	cyclin H [source: ZFIN; Acc: ZDB-GENE-050320-13]	zgc:114132
ENSDARG00000055989	tmem161b	protein_coding	−1	transmembrane protein 161B [source: NCBI gene; Acc: 406680]	id:ibd2207
ENSDARG00000009418	mef2cb	protein_coding	−1	myocyte enhancer factor 2cb [source: ZFIN; Acc: ZDB-GENE-040901-7]	si:ch211-202e12.2
ENSDARG00000059689	mblac2	protein_coding	−1	metallo-beta-lactamase domain containing 2 [source: ZFIN; Acc: ZDB-GENE-081104-313]	si:dkey-147l19.3
ENSDARG00000024687	polr3g	protein_coding	1	polymerase (RNA) III (DNA directed) polypeptide G [source: ZFIN; Acc: ZDB-GENE-081104-312]	si:dkey-147l19.2
ENSDARG00000024693	lysmd3	protein_coding	−1	LysM, putative peptidoglycan-binding, domain containing 3 [source: NCBI gene; Acc: 415194]	cb462
ENSDARG00000021137	adgrv1	protein_coding	1	adhesion G protein-coupled receptor V1 [source: NCBI gene; Acc: 415105]	gpr98
ENSDARG00000052690	arrdc3a	protein_coding	−1	arrestin domain containing 3a [source: ZFIN; Acc: ZDB-GENE-030131-2913]	arrdc3
ENSDARG00000067664	si:dkey-172m14.2	protein_coding	−1	si:dkey-172m14.2 [source: ZFIN; Acc: ZDB-GENE-060526-222]	
ENSDARG00000052693	si:dkey-172m14.1	processed_transcript	−1	si:dkey-172m14.1 [source: ZFIN; Acc: ZDB-GENE-060526-221]	
ENSDARG00000052695	nr2f1a	protein_coding	1	nuclear receptor subfamily 2, group F, member 1a [source: NCBI gene; Acc: 30418]	COUP(VI)
ENSDARG00000052697	fam172a	protein_coding	−1	family with sequence similarity 172 member A [source: NCBI gene; Acc: 393390]	si:dkey-172f14.1
ENSDARG00000077072	si:ch73-280o22.2	protein_coding	−1	si:ch73-280o22.2 [source: ZFIN; Acc: ZDB-GENE-141216-272]	gb:eb924149
ENSDARG00000102118	slf1	protein_coding	1	SMC5-SMC6 complex localization factor 1 [source: ZFIN; Acc: ZDB-GENE-141216-402]	si:ch73-280o22.1
ENSDARG00000076404	mctp1a	protein_coding	−1	multiple C2 domains, transmembrane 1a [source: ZFIN; Acc: ZDB-GENE-110125-3]	
ENSDARG00000079697	zgc:194908	protein_coding	−1	zgc:194908 [source: ZFIN; Acc: ZDB-GENE-081022-164]	zgc:194925
ENSDARG00000035193	fam81b	protein_coding	1	family with sequence similarity 81 member B [source: ZFIN; Acc: ZDB-GENE-130530-586]	zmp:0000000583
ENSDARG00000074314	ttc37	protein_coding	−1	tetratricopeptide repeat domain 37 [source: ZFIN; Acc: ZDB-GENE-110125-2]	
ENSDARG00000059711	nol6	protein_coding	1	nucleolar protein 6 (RNA-associated) [source: ZFIN; Acc: ZDB-GENE-030131-6294]	wu:fi43b02
ENSDARG00000059714	arsk	protein_coding	1	arylsulfatase family, member K [source: NCBI gene; Acc: 562412]	zgc:153019
ENSDARG00000024325	cert1a	protein_coding	1	ceramide transporter 1a [source: NCBI gene; Acc: 796455]	col4a3bp
ENSDARG00000052734	hmgcra	protein_coding	−1	3-hydroxy-3-methylglutaryl-CoA reductase a [source: ZFIN; Acc: ZDB-GENE-040401-2]	hmgcr1
ENSDARG00000052731	ankrd31	protein_coding	1	ankyrin repeat domain 31 [source: ZFIN; Acc: ZDB-GENE-050417-473]	zgc:113046
ENSDARG00000035198	gcnt4a	protein_coding	1	glucosaminyl (N-acetyl) transferase 4a [source: NCBI gene; Acc: 324510]	c2gnt3
ENSDARG00000059719	fam169aa	protein_coding	1	family with sequence similarity 169 member Aa [source: ZFIN; Acc: ZDB-GENE-060825-117]	im:7150681
ENSDARG00000078250	zgc:194398	protein_coding	1	zgc:194398 [source: ZFIN; Acc: ZDB-GENE-081022-111]	
ENSDARG00000067670	pomt1	protein_coding	1	protein-O-mannosyltransferase 1 [source: NCBI gene; Acc: 569769]	zgc:153731
ENSDARG00000032372	ccdc180	protein_coding	−1	coiled-coil domain containing 180 [source: ZFIN; Acc: ZDB-GENE-130530-582]	zmp:0000000579
ENSDARG00000067672	card9	protein_coding	−1	caspase recruitment domain family, member 9 [source: ZFIN; Acc: ZDB-GENE-060531-94]	si:dkey-1o2.6
ENSDARG00000067673	snapc4	protein_coding	−1	small nuclear RNA activating complex, polypeptide 4 [source: ZFIN; Acc: ZDB-GENE-030131-5794]	fi18h02
ENSDARG00000095515	entr1	protein_coding	−1	endosome associated trafficking regulator 1 [source: NCBI gene; Acc: 795251]	sdccag3
ENSDARG00000059722	ubac1	protein_coding	1	UBA domain containing 1 [source: ZFIN; Acc: ZDB-GENE-030131-9372]	fj67a11
ENSDARG00000063276	msh3	protein_coding	1	mutS homolog 3 (E. coli) [source: ZFIN; Acc: ZDB-GENE-060526-307]	si:dkey-56d12.1
ENSDARG00000002816	rasgrf2b	protein_coding	1	Ras protein-specific guanine nucleotide-releasing factor 2b [source: NCBI gene; Acc: 553520]	rasgrf2

### Antibody staining

Embryos were fixed at the indicated stage in 4% PFA for 1–2 h at room temperature, permeabilized in PBST (PBS + 0.5% Triton X-100) and blocked in PBST + 2% bovine serum albumin at room temperature. Antibodies were diluted in PBST + 2% BSA and incubated overnight at 4°C or at room temperature for 4 h. Samples were washed in PBST for 2 h between antibody applications and cleared overnight in 70% glycerol. Primary antibodies used and their concentrations were as follows: antiphospho-Histone H3 (1:200, Abcam, ab14955); antiactive Caspase-3 (1:700, BD Pharmingen, 559565); anti-Pax2a (1:200, GeneTex, GTX128127); anti-F59 (1:10, DSHB); anti-Znp-1 (1:200, DSHB); anti-Zn-5 (1:200, DSHB); anti-SV-2 (1:200, DSHB); anti-GFP (1:200, Invitrogen, A10262); Alexa Fluor 568-conjugated Phalloidin (1:500, Invitrogen, A12380); and Alexa Fluor 488-conjugated α-bungarotoxin (10 µg/ml, Invitrogen, B13422). Secondary antibodies used were as follows: Alexa Fluor 488-conjugated goat antirabbit (Invitrogen, A11008); Alexa Fluor 488-conjugated goat antimouse (Invitrogen, A11001); Alexa Fluor 488-conjugated goat antichicken (Invitrogen, A11039); Alexa Fluor 568-conjugated goat antirabbit (Invitrogen, A11011); and Alexa Fluor 568-conjugated goat antimouse (Invitrogen, A11004), all used 1:200. TO-PRO-3 iodide (1 µM, Invitrogen, T3605) was used to detect nuclei.

### RNA synthesis and injection

Capped RNA was synthesized using a *Not*I (NEB, R3189)-linearized pCS2 template (pCS2-EGFP-CAAX) and the mMessage mMachine SP6 kit (Invitrogen, AM1340). RNA was purified using the RNeasy Mini Kit (Qiagen, 74104) and ethanol precipitated. Around 150–250 pg of RNA was microinjected into 1-cell stage embryos.

### Imaging

For live or fixed whole-mount confocal imaging, embryos were embedded in 1.6% low-melt agarose (in E3 or PBS, respectively) in Pelco glass-bottom dishes (Ted Pella, 14027). Images were acquired using Zeiss LSM710 or LSM880 laser-scanning confocal microscopes using a 40× water-immersion objective (1.2 NA) and a 2.1 µm z-step (voxel size of 0.52 µm × 0.52 µm × 2.1 µm). One eye or one side of the trunk was imaged per embryo. Bright-field images and 3 dpf touch-response movies were acquired on an Olympus SZX16 stereomicroscope with a UC90 camera. Histological sections were imaged on an Olympus CX41 microscope with a DP25 camera using a 40× objective.

### Histology

Histology was performed using a previously published protocol (Nuckels and Gross 2007). Embryos were euthanized in tricaine (400 mg/100 ml, diluted further in E3) and fixed at 5 dpf in a solution of 4% formaldehyde, 2% glutaraldehyde, and 3% glucose in 0.1 M cacodylic buffer at 4°C. Tails were dissected prior to fixation to genotype embryos. Samples were subsequently fixed in 1% OsO_4_ at 4°C and dehydrated, then placed in LR White resin, and serial sectioned at 1 µm. Samples were stained with toluidine blue.

### Penetrance of mutant phenotypes

Embryos were screened under a dissecting stereomicroscope at 24 hpf for a visible lens; those without a visible lens were considered phenotypically mutant and separated for additional phenotyping. Embryos imaged under confocal microscope were used to quantify the penetrance of the optic cup defect, defined as the absence of space between the lens and retina; all other phenotypes were assayed under dissecting stereomicroscope. Paralysis was assayed at 3 dpf by touching the trunk/tail of each embryo with forceps; those that were touch-responsive (either twitched or swam away) were considered motile and not paralyzed. Heart edema and slowed heartbeat were assayed at 3 and 4 dpf. All embryos were assessed for heart edema (swelling around the heart), which was evident by 3 dpf and worsened by 4 dpf. Heart edema was found to only be present in genotypically mutant embryos. The heartbeat was counted in a 15-s interval for several embryos with edema (*n* = 7; range of beats/15 s: 6–23, mean = 18) or without edema (*n* = 4; range of beats/15 s: 40–49, mean = 43). All phenotyped embryos were then genotyped, and penetrance was calculated by dividing the number of genotyped *sco* mutants that presented with a given phenotype by the total number of *sco* mutants genotyped.

### Image analysis

Images were processed using Fiji (Schindelin et al. 2012), and 3D renderings were generated using FluoRender (Wan et al. 2012). Rendered images had the ectoderm digitally erased in Fiji prior to 3D visualization. Image quantifications were performed prior to genotyping, and one eye or one side of the trunk was imaged per embryo.

#### Quantification of activated caspase-3 and phospho-histone H3

Cells were tallied through the entire volume of the eye at 24 and 72 hpf in each 3D dataset using Fiji’s multipoint tool to label cells and avoid double counting. Only antibody-positive cells in the retina were quantified. A total of 24 hpf embryos were mounted dorsally, and 72 hpf embryos were mounted ventrally.

#### Quantification of Tg(cldnb:lyn-EGFP) and Pax2a

Images were laterally oriented in FluoRender and distal tissue was cropped away to visualize the mediolateral lens midpoint. A screenshot was captured in FluoRender and quantified in Fiji using the angle measurement tool, as schematized (Fig. 2, c and j). For *Tg(cldnb:lyn-EGFP)*, the rays of the angle were drawn at the nasal (anterior) margin of the retina and bordering the furthest *Tg(cldnb:lyn-EGFP)*-positive region. The embryos used in this experiment were double transgenic, in which the entire optic cup was labeled with *Tg(bactin2:EGFP-CAAX)*; only *Tg(cldnb:lyn-EGFP)*-expressing cells were double GFP+. This area was markedly brighter compared to the rest of the retina expressing only *Tg(bactin2:EGFP-CAAX)*, and the rays of this angle were drawn to encompass the brightest GFP+ domain. For Pax2a, the rays of the angle were drawn to encompass the temporal-most and nasal-most positive regions of antibody staining. The vertex for both measurements was positioned at the lens center. Angle measurements were performed twice and the median values were comparable for each round of analysis.

#### Quantification of retinal ganglion cell density

Using histological sections of 5 dpf embryos imaged at 40X magnification, the number of nuclei in the retinal ganglion cell layer (GCL) was counted in a 35 µm^2^ region of the central retina; this area represented about ∼one-third to one-half of the entire GCL in mutants. The mean and range were reported for sibling (*n* = 2) and mutant (*n* = 3) samples.

#### Quantification of Zn-5 (retinal GCL thickness)

The full volume of the 5 dpf larval eye was imaged ventrally and a single z-slice at the lens-midpoint was quantified. In Fiji, the width of the zn-5+ layer and the width of the total retina (as reported by nuclear TO-PRO-3 staining) were measured in three places, near the lateral edges of the nasal and temporal retina, and at the nasal-temporal midpoint of the retina, as schematized (Fig. 2, o and p). For GCL thickness, the three positions measured were averaged and reported for each embryo. For normalized GCL width (GCL: retina width), the width of the zn-5+ layer was divided by the total width of the retina, as measured at each location. GCL: retina width ratios were then averaged for each embryo.

#### Quantification of F59 (slow muscle fibers) and trunk vasculature

All trunk images were acquired at an anterior–posterior position dorsal to the yolk extension. For F59 (slow muscle fiber), the average length-to-displacement metric followed Chagovetz et al. (2019). Briefly, muscle fibers (8–14 fibers) in the same somite, imaged dorsal to the yolk extension, were measured from end-to-end using the straight-line tool (for displacement) and the segmented line tool (for length). A length-to-displacement ratio (length divided by displacement) was calculated for each fiber and then averaged for each embryo.

#### Quantification of znp-1 (motor neuron) axon length

All trunk images were acquired at an anterior–posterior position dorsal to the yolk extension. Data were 3D rendered in FluoRender, and the image was cut away to reveal caudal primary (CaP) motor neurons. A screenshot was captured in FluoRender and imported into Fiji, where the length of 3 CaP motor neurons was measured per embryo, as was the height of the trunk. Measurements were taken using the straight-line tool. The average motor neuron axon length was calculated per embryo and divided by the trunk height to yield a ratio.

#### Quantification of SV-2 and α-bungarotoxin colocalization

All trunk images were acquired at an anterior–posterior position dorsal to the yolk extension. Colocalization was performed on one side of the trunk: at 48 hpf, this included 12–24 z-slices and at 72 hpf, 14–32 z-slices, depending upon the embryo. Using the Coloc 2 macro in Fiji with a Costes threshold, a region that encompassed the trunk was drawn for each embryo, and the Pearson’s *R*^2^ colocalization coefficient between the two channels was reported for the specified region.

#### Quantification of superficial ocular vasculature

Images were laterally oriented in FluoRender and the number of dorsal ocular vessels (those in the upper half of the eye) were counted per embryo, as schematized (Fig. 5e).

### Statistical analysis

All analyses were performed and graphed in RStudio with the exception of estimation statistics (Fig. 4e). Statistical significance was determined using Welch’s *t*-test, from which a *P*-value of 95% or greater was considered significant. Box-and-Whisker plots were generated using the ggplot2 package. The band inside the box is the median. The upper and low “hinges” correspond to the first and third quartiles. The upper whisker extends from the upper hinge to the highest value within (1.5 × IQR), where IQR is the inter-quartile range. The lower whisker extends from the lower hinge to the lowest value within (1.5 × IQR). Data points outside of the ends of the whiskers are outliers. Estimation statistics were performed using the online platform https://www.estimationstats.com/#/ (Accessed: 2021 December 28), which output a Gardner–Altman estimation plot (Fig. 4e) (Ho et al. 2019). Five thousand bootstrap samples were taken; the confidence interval (CI) was bias-corrected and accelerated. The *P-*value reported was calculated from a two-sided permutation *t*-test, which tests the likelihood of observing the effect size, if the null hypothesis of zero difference is true. For each permutation *P-*value, 5000 reshuffles of the control and test labels were performed.

## Results

### Isolation of the *shutdown corner* mutant

**Fig. 1.**
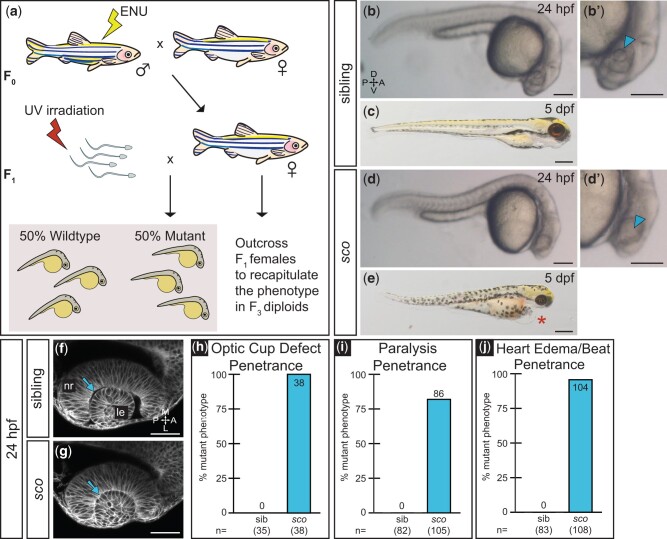
*Shutdown corner*, isolated in a haploid screen, exhibits a gross morphological defect of the optic cup. a) Haploid mutagenesis screen strategy. b, d) A defect in optic cup morphology is visible at 24 hpf. Lateral view of sibling b) and *sco* mutant d) diploid embryos under dissecting stereomicroscope. Zoomed views of sibling b′) and *sco* d′) lens regions (*arrowheads*), in which the lens is difficult to discern in the *sco* mutant. c, e) *sco* mutants exhibit heart edema (*asterisk*) and die around 5 dpf. f, g) Optic cup morphogenesis in live-imaged, membrane-labeled samples: a lens forms in sibling f) and *sco* mutants g) and is enwrapped by the developing retina in both. Dorsal view, single confocal section of 24 hpf *Tg(bactin2:EGFP-CAAX)* embryos. *Arrows* indicate the separation f) or apparent close association g) between the lens and neural retina. The *sco* mutant 24 hpf optic cup h), 3 dpf paralysis i), and 3 dpf heart edema/slowed heartbeat j) phenotypes are highly penetrant when screened on confocal or stereomicroscope. (h: sib = 0%, *sco* = 100%; i: sib = 0%, *sco* = 81.9%; j: sib = 0%, *sco* = 96.3%). A, anterior; L, lateral; le, lens; M, medial; nr, neural retina; P, posterior. Scale bar: b, b′, d, d′: 170 µm; c, e: 310 µm; and f, g: 50 µm.

To identify novel factors governing optic cup morphogenesis, we undertook a small-scale haploid mutagenesis screen (Fig. 1a). A haploid approach is beneficial in that it saves both time and space, and given that the optic cup forms early in development, at 24 hpf, we avoided many defects associated with haploid embryos (Westerfield 2000). Briefly, we mated ENU-treated wild-type F_0_ males to untreated females; eggs were collected from the subsequent F_1_ females and activated with UV-irradiated sperm to generate the haploid generation. Haploid embryos were screened under a stereomicroscope at 24 hpf to identify gross morphological optic cup defects. If a phenotype of interest was identified, the F_1_ mother was outcrossed to wild-type males, and the subsequent F_2_ generation was then incrossed to determine if diploid F_3_ offspring recapitulated the haploid phenotype.

In total, we screened ∼100 genomes and isolated one phenotype of interest that was reproduced in diploid embryos, reported here as *shutdown corner* (*sco*) (Fig. 1, b–h). In wild-type siblings, the lens was positioned centrally in the retina and could be resolved under stereomicroscope at 24 hpf (Fig. 1, b and b′, *arrowhead*). In contrast, in *sco* mutants, the lens appeared absent under stereomicroscope at this stage (Fig. 1, d and d′, *arrowhead*). Using the *Tg(bactin2:EGFP-CAAX)* transgene to visualize cell membranes, embryos were live-imaged at cellular resolution via confocal microscopy (Gordon et al. 2018). Doing so revealed that the lens was present in *sco* mutants and was enwrapped by the retina; both tissues appeared to have a grossly normal morphology (Fig. 1, f–g). Notably, a space was present between the retina and the lens in wild-type siblings (Fig. 1f, *arrow*), yet this space was absent in *sco* mutants (Fig. 1g, *arrow*). This novel phenotype was fully penetrant at 24 hpf in *sco* mutants (Fig. 1h). The close association between the lens and retina reminded us of a defensive cornerback in American (gridiron) football. An outstanding cornerback, who allows no separation between themself and the wide receiver they cover, is known as a “*shutdown corner.*”

In addition, *sco* mutants exhibited multiple tissue defects, including paralysis that was apparent by 3 dpf and was ∼80% penetrant (Fig. 1 and Supplementary Movies 1 and 2). Nearly all mutants (96%) developed heart edema (Fig. 1e, *asterisk*) and had a slow heartbeat by 3 dpf (Fig. 1j, and see *Materials andMethods* for details), and the homozygous mutation conferred lethality around 5 dpf (Fig. 1, c and e). Heterozygous carriers were adult viable and fertile.

### Optic cup patterning, retinal development, and cell proliferation and death defects arise in *sco* mutant eyes

**Fig. 2.**
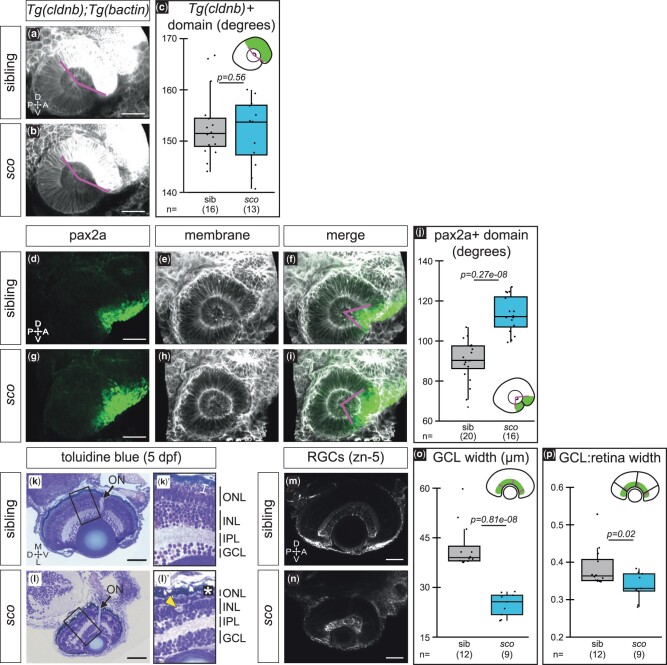
Optic cup patterning is partially altered and retinal defects arise in *shutdown corner.* a–c) Expression of *Tg(cldnb:lyn-EGFP)*, which labels the nasal (anterior) hemisphere of the optic cup at 24 hpf. 3D-rendered, lateral view of *Tg(cldnb:lyn-EGFP);Tg(bactin2:EGFP-CAAX)* embryos. a, b) *Magenta lines* demarcate *Tg(cldnb:lyn-EGFP)*-positive region. c) Quantification of *Tg(cldnb:lyn-EGFP)*-positive domain per embryo. d–j) Antibody staining for pax2a, a ventral marker, at 24 hpf. 3D-rendered, lateral views. Pax2a (d, g; *green*), cell membranes (e, h; *grayscale*, EGFP-CAAX), merge f, i). *Magenta lines* f, i) demarcate pax2a-positive region. j) Quantification of the pax2a-positive domain per embryo. k, l) 5 dpf histological sections stained with toluidine blue (imaged at 40x, sections at similar depth based on presence of optic nerve; sibling *n* = 2, *sco n* = 3) with zoomed views of the retina (k′, l′). *Arrows*, optic nerve; *bracket*, photoreceptor outer segments k′); *asterisk*, missing photoreceptor outer segments l′); *arrowhead*, potential cell death l′). m–p) RGCs (zn-5 staining) at 5 dpf. Ventral view, single confocal section from 3D datasets of antibody-stained samples. o, p) Quantification of GCL thickness, presented as the raw width o) and the width normalized to the total width of the retina p). Width measurements were taken at three places in each retina, at a nasal, temporal, and nasal-temporal midpoint; each point represents the average GCL width (raw or normalized) per embryo. *ON*, optic nerve; *ONL*, outer nuclear/photoreceptor layer; *INL*, inner nuclear layer; *IPL*, inner plexiform layer; *GCL*, ganglion cell layer; *RGC*, retinal ganglion cell; A, anterior; D, dorsal; L, lateral; M, medial; ON, optic nerve; P, posterior; V, ventral. Scale bar, 50 µm.

Given the unusual *sco* eye phenotype, we first asked whether optic cup development occurred normally by assaying anteroposterior (nasal-temporal) and dorsoventral patterning. Defects in either could indicate perturbations in cell signaling and/or cell movements. The transgenic line *Tg(cldnb:lyn-EGFP)* labels the anterior/nasal hemisphere of the optic cup (Picker et al. 2009), thus we imaged *sco* mutant and wild-type sibling *Tg(cldnb:lyn-EGFP);Tg(bactin2:EGFP-CAAX)* optic cups at 24 hpf (Fig. 2, a–c). The *Tg(bactin2:EGFP-CAAX)* transgene enabled us to visualize the whole optic cup and provided spatial context for the *Tg(cldnb:lyn-EGFP)*-positive domain. We quantified the domain of the optic cup expressing *cldnb:lyn-EGFP* as an angle measurement, where each ray was drawn at the two margins of the double GFP-positive domain (Fig. 2c, schematic). This metric revealed no significant difference in the size of the anterior/nasal hemisphere between wild-type siblings and *sco* mutants (Fig. 2c), indicating that anteroposterior (nasal-temporal) patterning was preserved. To assess dorsoventral patterning, we performed antibody staining against pax2a, an established ventral marker (Fig. 2, d–j) (Lee et al. 2008; Sedykh et al. 2017). We quantified the optic cup domain with pax2a-positive cells by again using an angle measurement, where each ray was drawn to encompass the pax2a-positive region (Fig. 2j, schematic). There was a significant increase in the pax2a-positive domain in the *sco* mutant optic cup (Fig. 2j), indicating that dorsoventral patterning, unlike anteroposterior patterning, was disturbed in *sco* mutants.

The cells that comprise the 24 hpf optic cup retinal domain begin to differentiate into neuronal cell types ∼30 hpf, first with retinal ganglion cells (RGCs) in the ventronasal aspect of the optic cup. Because the pax2a-positive domain in *sco* was expanded, we were curious if this early dorsoventral patterning defect translated into later retinal defects. We carried out histology to assay retinal lamination at 5 dpf (Fig. 2, k and l), the latest stage we can successfully rear mutants. The lens appeared free of irregularities, indicating that cataracts did not form by this stage. Retinal lamination also appeared to occur normally, with identifiable and organized retinal layers (Fig. 2, k′ and l′), including the GCL, inner plexiform layer (IPL), inner nuclear layer (INL), and outer nuclear (photoreceptor) layer (ONL). However, the inner and outer nuclear layers appeared thinner in *sco* mutants and, while they were present, photoreceptors appeared to lack outer segments (compare Fig. 2k′, *bracket*, to Fig. 2l′, *asterisk*). The *sco* mutant eye as a whole appeared smaller and there was evidence of potential cell death at this stage (Fig. 2l′, *arrowhead*). It was observed that the RGC layer in particular exhibited an irregular shape and appeared more densely packed. We examined cell density by counting the number of nuclei within a 35-µm^2^ central region of the GCL and found that cell packing was increased in *sco* mutants [means: sibling = 21 (range: 20–21); mutant = 36 (range: 29–43)]. We also carried out antibody staining using zn-5 to specifically label and examine the RGC population (Fig. 2, m–p). While the robust labeling of zn-5 indicated that differentiation was intact, the thickness of the GCL was significantly reduced in mutants (Fig. 2o). Because the *sco* mutant eye was visibly smaller, we normalized GCL width to the total width of the retina; this analysis revealed GCL thickness was subtly but still significantly decreased in mutants (Fig. 2p; means: sibling = 0.39; mutant = 0.34). These data suggest that the optic cup patterning defect in *sco* did not manifest in failure of retinal lamination and/or differentiation, although it is also clear that the *sco* retina is abnormal in other ways.

**Fig. 3.**
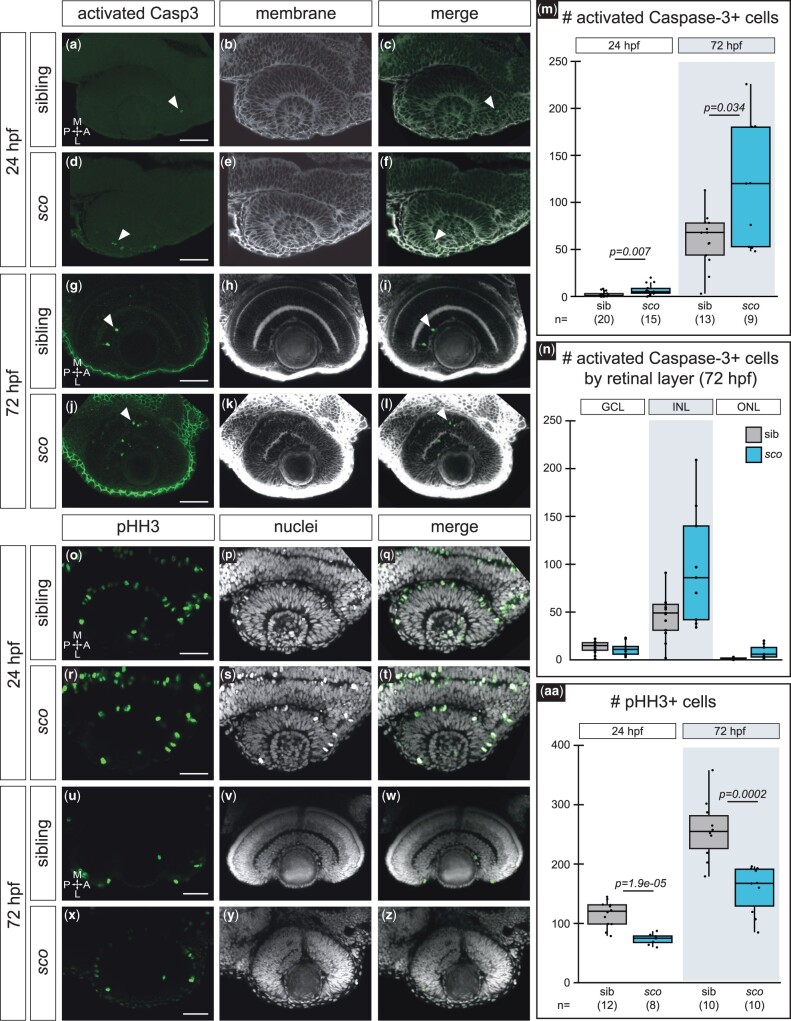
Cell death and proliferation are affected in the *shutdown corner* retina. a–n) Activated caspase-3-positive cells at 24 hpf a–f) and 72 hpf g–l) in siblings and *sco* mutants. Dorsal (24 hpf) or ventral (72 hpf) view, single confocal section from 3D datasets of antibody-stained *Tg(bactin2:EGFP-CAAX)* samples. Activated caspase-3 (a, d, g, j; *green*), cell membranes (b, e, h, k; *grayscale*, EGFP-CAAX), merge (c, f, i, l). *Arrowheads* (a, c, d, f, g, i, j, l), activated caspase-3 positive cells in the retina. m) Quantification of cells positive for activated caspase-3 in the retina, per embryo. n) Quantification of cells positive for activated caspase-3 at 72 hpf in each retinal layer, per embryo. o–aa) Phospho-histone H3 (pHH3)-positive cells at 24 hpf o–t) and 72 hpf u–z) in siblings and *sco* mutants. Dorsal (24 hpf) or ventral (72 hpf) view, single confocal section from 3D datasets of antibody-stained samples. pHH3 (o, r, u, x; *green*), nuclei (p, s, v, y; *grayscale*, TO-PRO-3), and merge (q, t, w, z). aa) Quantification of cells positive for pHH3 in the retina, per embryo. *GCL*, ganglion cell layer; *INL*, inner nuclear layer; *ONL*, outer nuclear layer; A, anterior; L, lateral; M, medial; P, posterior. Scale bar, 50 µm.

Because we captured potential incidences of cell death in the 5 dpf *sco* mutant retina, we sought to determine whether cell proliferation or cell death was perturbed in the *sco* retina at optic cup stage (24 hpf), and at 72 hpf, when retinal cell types are differentiating but before *sco* mutants die. Using an activated caspase-3 antibody (Fig. 3, a–n), which labels apoptotic cells, we observed increased apoptotic cell death in *sco* mutant retinas at both 24 and 72 hpf compared to wild-type siblings (Fig. 3, d–f,j–m, *arrowheads*). At 72 hpf, the cell death was mostly in the inner nuclear layer in wild-type siblings. In *sco* mutants, cell death was increased in both the inner and outer nuclear layers, but not the GCL (Fig. 3n). Using a phospho-histone H3 antibody, which detects cells in the G2/M phase of the cell cycle, we also found a decrease in cell proliferation in *sco* mutant retinas compared to wild-type siblings (Fig. 3, o–aa). Our observation of increased cell death at both 24 and 72 hpf is consistent with the possible cell death found via histology at 5 dpf (Fig. 2l′). Furthermore, the small size of the 5 dpf *sco* mutant eye could possibly be explained by these observations of increased cell death and decreased proliferation. Additional study would be necessary to test this and to rule out other potential mechanisms.

These data demonstrate that while optic cup morphology and retinal organization were maintained in *sco*, specific aspects of eye development were disrupted: proliferation was decreased, and apoptotic cell death was increased. The wider pax2a-positive domain also indicates that either cell signaling and patterning defects are present in *sco* mutants, or the cell movements underlying optic cup formation are altered, or a combination of both. Specific retinal layers, including RGCs, were affected in *sco* mutants as well, although it is unclear if that can be explained by the cell death and cell proliferation phenotypes, or patterning alterations, or both.

### *sco* mutants are paralyzed prior to death

**Fig. 4.**
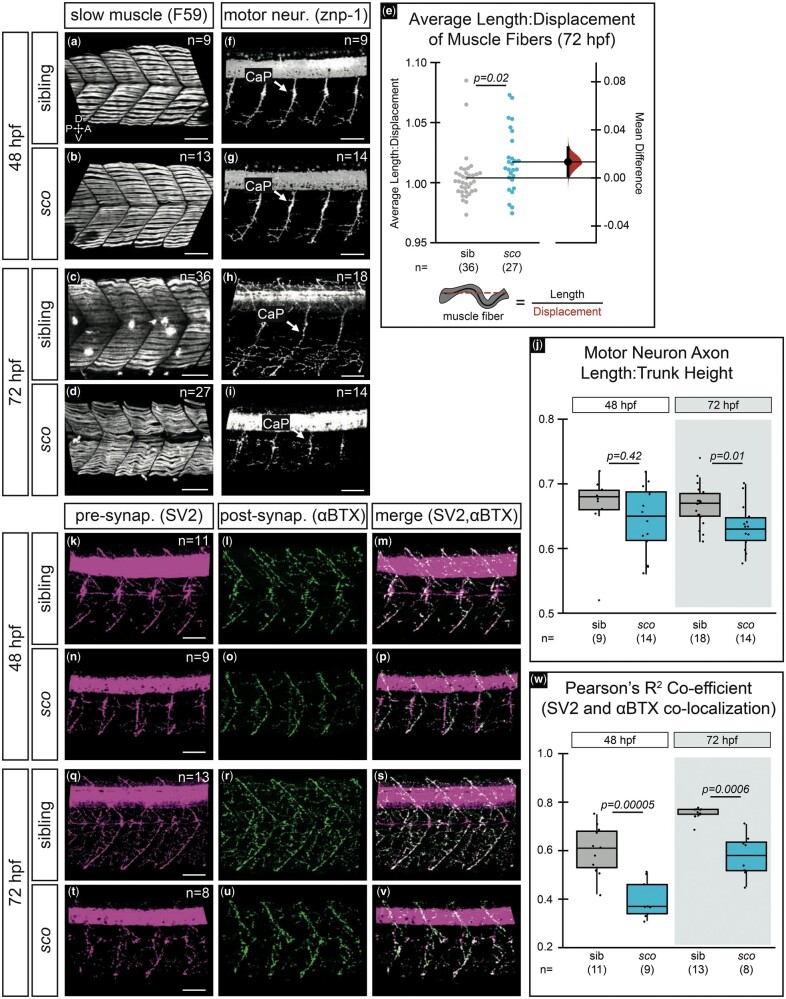
*Shutdown corner* has defective slow muscle fibers, motor neurons, and neuromuscular junctions. Slow muscle fibers (F59 staining) at 48 hpf a, b) and 72 hpf c, d) in siblings and *sco* mutants. e) Quantification of slow muscle fiber length-to-displacement ratio at 72 hpf presented as a Gardner-Altman estimation plot. *Left dot plots*, each data point represents an average of 8–14 fibers per single embryo measured as schematized. *Right bootstrap sampling distribution*, the mean difference between siblings and mutants is 0.0133 (95% CI: 0.00188, 0.0252). Mean difference depicted as a dot; 95% CI indicated by ends of vertical error bar reflects the effect size. f–j) Motor neurons (znp-1 staining) at 48 f, g) and 72 hpf h, i) in siblings and *sco* mutants. j) Quantification of motor neuron axon length normalized to trunk height. Three axons were measured per embryo; each point represents the average length ratio per embryo. k–w) Presynaptic terminals (SV-2 staining) and postsynaptic terminals (α-bungarotoxin or αBTX staining) at 48 hpf k–p) and 72 hpf q–v). SV-2 (k, n, q, t; *magenta*), αBTX (l, o, r, u; *green*), merge m, p, s, v). w) Quantification of SV-2 and αBTX colocalization at 48 and 72 hpf for one side of the trunk, per embryo. All images are 3D rendered, lateral views of the trunk region, dorsal to the yolk extension. Sample size (*n*) in images. A, anterior; CaP, caudal primary motor neuron; D, dorsal; P, posterior; V, ventral. Scale bar, 50 µm.

In conjunction with our work characterizing the eye, we observed that *sco* mutants have heart edema and some are slow or fail to hatch (data not shown). When swirled in a Petri dish, wild-type larvae swam to the periphery, yet 3 dpf mutants pooled in the middle of the dish. By 3 dpf, *sco* mutants also stopped responding to touch (Supplementary Movies 1 and 2) and appeared paralyzed; this phenotype is ∼80% penetrant (Fig. 1i). To try to determine the cellular basis of this phenotype, we carried out antibody staining for slow muscle fibers (Fig. 4, a–d) and motor neurons (Fig. 4, f–i). Because some *sco* mutants moved prior to 3 dpf (data not shown), we assayed for differences between 48 hpf (2 dpf) and 72 hpf (3 dpf). Staining for slow muscle fibers using the F59 antibody to label myosin heavy chain revealed that slow muscles initially appeared identical between wild-type siblings and *sco* mutants at 48 hpf (Fig. 4, a and b); however, by 72 hpf, these fibers developed an abnormally wavy morphology in *sco* mutants compared to wild-type siblings (Fig. 4, c and d). We quantified this waviness as a length-to-displacement ratio, where a value of 1.0 corresponds to a flat line (Fig. 4e) (Chagovetz et al. 2019). Wild-type siblings clustered near a perfectly straight line (mean = 1.00), while *sco* mutant embryos had a mild, but statistically significant greater length-to-displacement ratio [unpaired mean difference between sibling and *sco* mutants = 0.0133 (95% CI: 0.00188, 0.0252); *P* = 0.02]. Although the difference in these ratios appeared quantitatively small, similar quantitative differences were observed in other paralyzed zebrafish mutants (Chagovetz et al. 2019).

Staining with znp-1, an antibody against synaptotagmin-2, allowed us to examine differences in motor neuron development at these stages (Fig. 4, f–i). At 48 hpf, caudal primary (CaP) motor neuron axons were present and had a similar shape between genotypes at each stage, and there were no ectopic branches or truncated axons evident in mutants. However, when CaP axon length was measured and normalized to trunk height (Fig. 4j), axons were found to be to proportionally shorter at 72 hpf compared to wild-type siblings (Fig. 4, h–j). Given this defect in motor neurons, we also determined the integrity of neuromuscular junctions by assessing the colocalization between pre- and postsynaptic proteins (Fig. 4, k–w). In a normal functioning neuromuscular junction, there should be a close association between these proteins (Lupa and Hall 1989; Boon et al. 2009). We performed antibody staining against the presynaptic protein, synaptic vesicle protein 2 (SV-2), and postsynaptic acetylcholine receptors, as labeled by α-bungarotoxin (αBTX). There was strong staining against both proteins in siblings and *sco* mutants, but we found *sco* mutants had significantly reduced colocalization of SV-2 and αBTX at both 48 and 72 hpf (Fig. 4w), indicating a neuromuscular junction defect was present by 48 hpf and persisted through 72 hpf. These data suggest *sco* mutants may exhibit a neuromuscular defect earlier than 72 hpf that is more subtle than paralysis. Considering these results together, previous work has shown that myofibril organization is dependent upon muscle contraction (Brennan et al. 2005), thus the *sco* mutant paralysis phenotype may be primarily attributed to a neuromuscular junction and/or primary motor neuron defect that also manifested in less organized slow muscle fibers. Additional functional analysis may reveal further nuances within the *sco* mutant paralysis phenotype.

### Vasculature is disrupted in the *sco* mutant eye

**Fig. 5.**
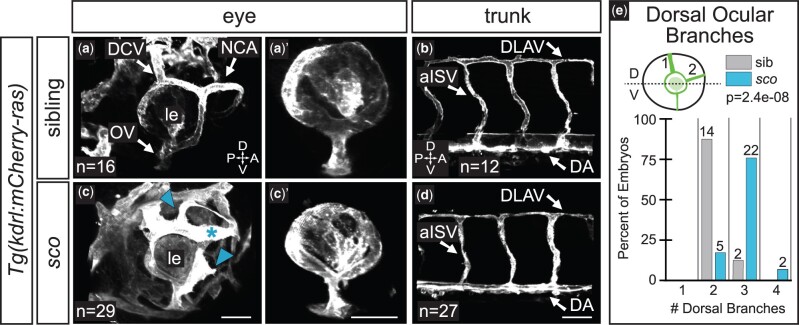
Ocular vasculature but not trunk vasculature is disrupted in *shutdown corner.* Ocular a, c) and trunk b, d) vasculature at 48 hpf [*grayscale*, *Tg(kdrl:mCherry-ras)*]. 3D renderings, lateral views. a′, c′) Hyaloid network; superficial vasculature cropped away in FluoRender. c) *Arrowheads* indicate ectopic branches; *asterisk* indicates a morphologically abnormal vessel. e) Quantification of the number of superficial ocular vessels in the dorsal half of the eye at 48 hpf. Dashed line in schematic demarcates the dorsal (D) and ventral (V) halves of the eye. Sample size (*n*) in images. a–d) Arrows pair labels with corresponding vessels. *le*, lens; *DCV*, dorsal ciliary vein; *NCA*, nasal ciliary artery; *OV*, optic vein; *DLAV*, dorsal longitudinal anastomosing vessel; *aISV*, arterial intersegmental vessel; A, anterior; P, posterior; D, dorsal; V, ventral. Scale bar, 50 µm.

Our phenotypic analysis primarily focused on the eye and the trunk, and we became curious if we could detect defects in other tissues in these regions. To this end, we evaluated vasculature development in both the eye and trunk at 48 hpf (Fig. 5, a–d). The ocular vasculature was present but aberrant. Hyaloid vasculature was intact (Fig. 5, a′ and c′), as well as the optic vein (OV), and vessels that comprise the superficial vasculature (Fig. 5, a and c), including the dorsal ciliary vein (DCV) and nasal ciliary artery (NCA). However, in *sco* mutants, there were ectopic superficial branches (Fig. 5c, *arrowheads*) and some superficial vessels had an abnormal morphology (Fig. 5c, *asterisk*). Most mutants (*n* = 24/29) had 1–2 more dorsal vessels than wild-type siblings (Fig. 5e). In contrast to the eye, we found that trunk vasculature (Fig. 5, b and d) appeared normal in *sco* mutants, with key vessels present, including the dorsal longitudinal anastomosing vessel (DLAV), arterial intersegmental vessel (aISV), and the dorsal aorta (DA). In total, in *sco* mutants, major vessels in the trunk and the eye formed by 48 hpf, but aberrances arose specifically in the superficial ocular vasculature.

### *Shutdown corner* is a 10-Mb deletion on chromosome 5

**Fig. 6.**
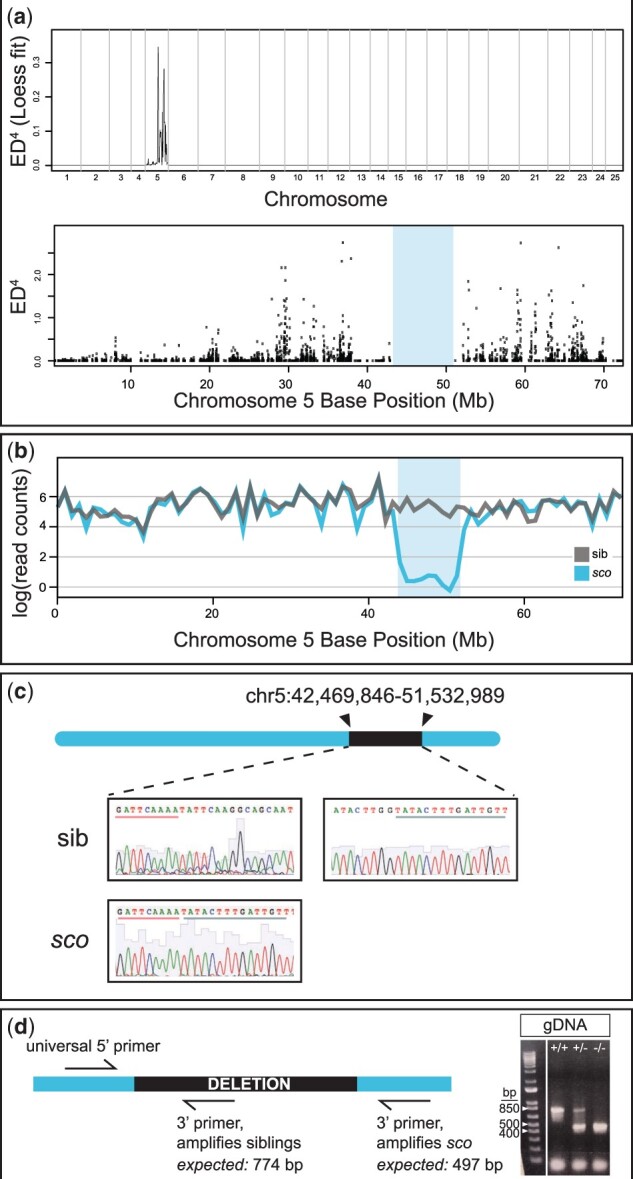
*Shutdown corner* harbors a large deletion on chromosome 5. a) *Top*, Genome wide Euclidean distance scores, fit with a local regression (LOESS) curve and raised to the fourth power to decrease noise. Note the large feature on chromosome 5. *Bottom*, Raw Euclidean distance scores for chromosome 5; note the gap between ∼40 and 50 Mb (*shaded region*). b) Comparison of RNA-sequencing read counts, fit with a local regression, between wild-type sibling (gray) and *sco* mutants (blue). There is a dramatic decrease in mapped reads in the ∼40–50 Mb region. c) Sanger sequencing of a wild-type sibling and *sco* mutant confirms the *shutdown corner* breakpoint. d) Schematic of genotyping primers and image of PCR products from genomic DNA (gDNA); using the three primers, the wild-type amplicon is 774 bp and the *sco* amplicon is 497 bp; heterozygotes have both a wild-type and *sco* mutant amplicon. Reference band sizes in ladder are annotated. Bp, base pair.

To identify the *sco* genetic lesion, we utilized the program MMAPPR, an RNA-sequencing-based platform designed to identify putative mutated loci from samples pooled by phenotype (Hill et al. 2013). We phenotyped embryos as wild-type siblings or *sco* mutants based on their 24 hpf optic cup phenotype, and confirmed the phenotyping by examining the 3 dpf paralysis phenotype. We performed RNA-sequencing at 3 dpf and input these results into the MMAPPR pipeline. We anticipated MMAPPR to indicate a sharp peak, suggestive of a mutation at a single locus, but instead observed a broad feature on chromosome 5 (Fig. 6a, top). Looking more closely at chromosome 5 (Fig. 6a, bottom), MMAPPR showed an absence of data between ∼40 and 50 Mb (Fig. 6b). Further analysis of the RNA-seq data confirmed that this was due to a complete lack of RNA-sequencing reads aligned to this region of the genome specifically in the mutant pool; reads were present for this region in the sibling pool. Together, these data suggested that *sco* carries a deletion on chromosome 5. This was confirmed by PCR of genomic regions inside and outside of the predicted deletion interval. To precisely identify the deleted region, we performed a series of PCR experiments on genomic DNA to “walk” closer to the breakpoint until the breakpoint was successfully cloned and the exact breakpoint identified by Sanger sequencing (Fig. 6c, sequencing shown is from wild-type sibling and *sco* mutant embryos).

The deletion spans the interval of chr5:42,469,846–51,532,989, in the region initially suggested by MMAPPR [Fig. 6, a (bottom) and b] and encompasses 89 annotated genes (Table 1). Therefore, we refer to this mutant line as *Df(Chr05:sco)^z207^.* Similar to how the mutation was eventually identified, genomic PCR using primers positioned inside and outside of the deletion can be used to genotype *sco* mutants or heterozygous and homozygous wild-type siblings (Fig. 6d). To our knowledge, this is the first study to demonstrate that MMAPPR can successfully identify large deletions. Furthermore, *shutdown corner* is a large chromosomal deletion, few of which have been reported in zebrafish.

## Discussion

We describe here a haploid screen and isolation of a ∼10-Mb deletion mutant, *shutdown corner* [*Df(Chr05:sco)^z207^*], which exhibits multiple tissue defects. Although *sco* was originally isolated for its unusual 24 hpf optic cup defect, other phenotypes, including paralysis and heart edema, were readily observed. We have reported here various phenotypes in *sco* mutants, and we anticipate other defects may become apparent with more thorough and focused analyses.

Several eye phenotypes of interest were recovered in *sco*, including an apparent lack of space between the lens and retina, a defect in the superficial ocular vasculature, impaired dorsal-ventral patterning, and altered GCL thickness and cell density. Space between the lens and retina has been observed across vertebrates (Hunt 1961; Silver and Wakely 1974; Jackson 1976; McAvoy 1980), but the precise functional significance of this separation, and its loss, is unknown. Interestingly, the ocular hyaloid vasculature that develops behind the lens formed normally by 48 hpf in *sco*; rather, the superficial vasculature developed ectopic branches and some vessels exhibited an abnormal morphology. It seems formation and/or maintenance of the lens-retina space is dispensable for lens development, as the lens was morphologically normal by 5 dpf, but an effect on retinal development cannot be ruled out. Expansion of the ventral domain of the *sco* optic cup may also partially underlie the GCL thickness and cell density defects, as RGCs initiate differentiation in the ventronasal region of the eye soon after optic cup stage. Whether the increased ventral domain in *sco* relates to the RGC development defects is unclear, although it is intriguing to consider how an early defect in morphogenetic cell movement and/or cell signaling events might impair subsequent RGC development. Ultimately, the many developmental programs underlying eye development must be integrated to yield a functional organ, and specific defects in one or multiple processes could directly and/or indirectly impair other subsequent events. The *sco* mutant eye raises questions not only about how cells within the retina develop and differentiate, but also about the reciprocal interactions between the retina and extrinsic tissues like the lens and vasculature.

As a first approach to assessing the genes lost within the interval, we carried out gene ontological analysis to determine if there was any enrichment in biological process, molecular function, or cellular localization within our 89 gene list. Using the PANTHER Classification System (Mi et al. 2019), we tested for enrichment using Fisher’s exact test and corrected for false discovery rate, which revealed no significant enrichment (data not shown). This was not surprising, as gene clusters have only been reported in zebrafish for the major histocompatibility complex and HoxA (HoxAa and HoxAb) (Howe et al. 2013). When individually examined, several genes within the deletion interval could explain some of the phenotypes we observed. The photoreceptor defect in *sco* may be explained by loss of *adgrv1* and/or *poc5*, two genes implicated in the degenerative condition retinitis pigmentosa (Ebermann et al. 2010; Weisz Hubshman et al. 2018). Knockout of the gene *rasa1* was shown to impair mouse retinal vasculature, suggesting it could be involved in the ocular vasculature phenotype (Chen et al. 2019). The *sco* paralysis phenotype could be tied to loss of *gas1a*, a factor involved in skeletal myogenesis (Leem et al. 2011), and/or *smn1*, which is associated with skeletal muscle atrophy, caused by progressive loss of motor neurons (Fallini et al. 2012; Hao Le et al. 2017). Neuromuscular junction defects, specifically decreased colocalization of pre- and postsynaptic proteins, were also found in zygotic *smn1* zebrafish mutants, indicating this gene may underlie the *sco* paralysis phenotype (Boon et al. 2009). Interestingly, maternal-zygotic *smn1* zebrafish mutants exhibited a more severe phenotype, with truncated motor neurons that developed fewer branches (Hao et al. 2013), underscoring a potential contribution of maternally supplied smn1 in *sco*. Several factors in the deletion interval are expressed in the heart and may contribute to the slow heartbeat observed in *sco* mutants. Mutations in *vcan* are embryonic lethal in mouse and medaka due to impaired heart development (Mjaatvedt et al. 1998; Mittal et al. 2019), and another factor, *mef2cb*, has also been associated with heart defects in zebrafish (Hinits et al. 2012). Although we did not evaluate it in this study, edema can also indicate kidney dysfunction, and knockdown of the gene *iqgap2* was reported to impair zebrafish glomerular formation and result in heart edema (Sugano et al. 2015). Despite these intriguing connections, individual contribution of any one gene to the *sco* phenotype would be difficult to parse within the scope of this study. Synergism between genes must also be considered, as should the potential loss of regulatory elements that act on genes outside of the deletion interval. Maternal deposition of transcripts and/or proteins may modulate all of these genetic processes as well.

Despite these caveats, we believe that *shutdown corner* may be of interest and use to the zebrafish community at large, potentially as a starting point for other studies. Large deletions have a history of utility in systems like *Drosophila*, where significant work has gone into generating a series of deletion mutants (Ryder et al. 2007; Wright et al. 2010; Cook et al. 2012). These mutants have aided gene mapping efforts and enabled studies into genetic interactions. An analogous resource in zebrafish does not exist and, to the best of our knowledge, *shutdown corner* is the largest deletion reported to date. Advances in CRISPR-based mutagenesis have made generating deletions a feasible goal, but currently the largest reported engineered deletion is less than 300 kb in size (HoshijiMa et al. 2019; Kim and Zhang 2020; Tromp et al. 2021).

In addition, reverse genetics in zebrafish using engineered putative loss-of-function mutants have commonly failed to present with phenotypes, often in contrast to previously observed morpholino-mediated knockdown phenotypes (Kok et al. 2015; Stainier et al. 2017). It was demonstrated that compensatory mechanisms may be in place that mask loss-of-function phenotypes, in zebrafish as well as other model systems (Rossi et al. 2015; Lalonde et al. 2017; El-Brolosy et al. 2019; Ma et al. 2019). To avoid triggering this machinery, the zebrafish field is moving toward mutagenesis strategies that ablate transcription. Because the ∼10-Mb interval on chromosome 5 in *sco* is entirely lost, we anticipate that all 89 annotated genes in the interval are a complete loss-of-function and, as they are not transcribed, do not trigger compensatory mechanisms. We have shown that *shutdown corner* exhibits many phenotypes which require additional study, and we anticipate other defects may be captured through further analysis. Therefore, *sco* may be useful to researchers who are mapping a mutation, interested in understanding genetic interactions, and/or wishing to obtain an initial assessment of whether a gene in the interval may be involved in their biology of interest.

It is also surprising that we isolated a ∼10-Mb deletion mutant from an ENU-based screen. ENU is best known for inducing point mutations, which raises the possibility that the mutation may have existed in the background of our fish population. Despite the small-scale nature of our screen, our successful identification of a mutant with an unusual optic cup defect underscores the need to carry out screens specific to optic cup morphogenesis and suggests that other phenotypes may be identified with further screening. Our combination of a haploid mutagenesis screen with contemporary computational methods led to our identification of a novel mutant, *shutdown corner* [*Df(Chr05:sco)^z207^*], a ∼10-Mb deletion mutant, which shows potential for this method’s utility in the zebrafish community.

## Data availability

The data underlying this article are available in the Sequence Read Archive at https://www.ncbi.nlm.nih.gov/sra (Accessed: 2021 December 29) and can be accessed with PRJNA790108.

Supplemental material is available at *G3* online.

## Supplementary Material

jkab442_Supplemental_Movie_Legends

jkab442_Supplementary_Movie_1

jkab442_Supplementary_Movie_2
