# Supplementary material for: Shutdown corner, a large deletion mutant isolated from a haploid mutagenesis screen in zebrafish
Source: G3 (Bethesda). 2021 Dec 23;12(3):jkab442. doi: 10.1093/g3journal/jkab442 (PMC9210284; doi:10.1093/g3journal/jkab442)
Supplement: jkab442_Supplemental_Movie_Legends [file jkab442_supplemental_movie_legends.docx]

**Movie 1. Wildtype siblings are touch responsive and swim away at 3 dpf.**

**Movie 2. *shutdown corner* is paralyzed and does not respond to touch at 3 dpf.**
